# Growth of human breast cancers in *Peromyscus*

**DOI:** 10.1242/dmm.031302

**Published:** 2018-01-01

**Authors:** Vimala Kaza, Elena Farmaki, Amanda Havighorst, Janet Crossland, Ioulia Chatzistamou, Hippokratis Kiaris

**Affiliations:** 1 Peromyscus Genetic Stock Center, University of South Carolina, SC 29208, USA; 2 Department of Drug Discovery and Biomedical Sciences, College of Pharmacy, University of South Carolina, SC 29208, USA; 3 Department of Pathology, Microbiology and Immunology, University of South Carolina School of Medicine, SC 29208, USA

**Keywords:** Outbred, Mammary cancer, Diversity, Heterogeneity

## Abstract

Modeling breast cancer in general and hormone-sensitive breast cancer, in particular in mice, has several limitations. These are related to the inbred nature of laboratory mice, and do not allow adequate appreciation of the contribution of the host's genetic heterogeneity in tumor growth. In addition, the naturally low estrogen levels of mice makes estradiol supplementation obligatory for tumor growth. Here, we show that *Peromyscus californicus*, following cyclosporine-mediated immunosuppression, supports the growth of both MDA-MB-231 estrogen-independent and MCF7 estrogen receptor-positive breast cancers without exogenous estradiol supplementation. Tumor growth was inhibited by fulvestrant or letrozole, confirming that MCF7 xenografts remain hormone dependent *in vivo* and suggesting that *P. californicus* can be used as an alternative to conventional mice for the study of hormone-sensitive breast cancer. The fact that *Peromyscus* stocks are outbred also facilitates the study of breast cancer in genetically heterogenous populations.

## Abstract (precis)

**Summary:** Outbred stocks of *Peromyscus californicus* (California mice), upon pharmacological immunosuppression, provide an alternative to conventional inbred mice models, and can support the growth of hormone-insensitive and hormone-sensitive human breast cancers.

## INTRODUCTION

The use of mice (genus *Mus*) as a cancer model has several limitations. The inbred nature of conventional laboratory mice limits our ability to adequately appreciate the impact of the host's genetic diversity in tumorigenesis and drug efficacy. In addition, in the context of estrogen-sensitive breast cancer, low endogenous estrogen levels render supplementation of animals with exogenous estrogen necessary to sustain the growth of hormone-sensitive breast cancers. Although estrogen supplementation stimulates tumorigenesis, this treatment has several complications, including toxicity to the host and maintenance of supraphysiological estradiol (E2) levels throughout the course of the experiment (Gakhar et al., 2009; Pearse et al., 2009; Chatzistamou and Kiaris, 2016). Furthermore, major classes of breast cancer therapeutics that target the hosts' cellular processes, such as the aromatase inhibitors, cannot be evaluated in intact breast cancer cells unless they have been manipulated to overexpress and, thus, depend on aromatase for their growth. In addition, these models must be supplemented with an androgen substrate such as androstenedione, further reducing their relevance to clinical breast cancer (Long et al., 2004; Brodie et al., 2010; Lønning and Eikesdal, 2010).

The identification of mammals that can sustain the growth of estrogen-dependent cancers *in vivo* without exogenous E2 supplementation could overcome a major challenge in modeling hormone-sensitive breast cancers. Ideally, such models are also outbred, facilitating the study of human breast cancer in genetically heterogenous hosts, simulating better the human populations in which the disease develops. Considering earlier studies reporting elevated E2 levels in their sera, we hypothesized that animals of the genus *Peromyscus* might support the growth of estrogen-dependent cancers without exogenous E2 supplementation (Chatzistamou and Kiaris, 2016; Steger et al., 1980).

## RESULTS

### MDA-MB-231 tumor growth

In order to overcome xenograft rejection owing to the lack of naturally existing immunoincompetent *Peromyscus*, we treated animals daily with 50 mg/kg cyclosporine A (CsA) subcutaneously (s.c.). CsA has been shown to cause immunosuppression in mice and rats and permits the growth of human cancer xenografts *in vivo* (Goodman et al., 1987; Fingert et al., 1984; Bennett et al., 1985). To confirm that CsA sufficiently immunosuppresses *Peromyscus*, 5- to 8-month-old female *Peromyscus* *leucopus* and *Peromyscus* *californicus* (*n*=5 for each) obtained from the Peromyscus Genetic Stock Center (University of South Carolina) were implanted orthotopically with the highly aggressive triple-negative breast cancer cells MDA-MB-231 admixed with human fetal foreskin fibroblasts (HFFF2, 3 million each). MDA-MB-231 cells were selected for these initial studies because they are highly tumorigenic, regardless of host E2 levels. In view of the fact that *Peromyscus* live about three times longer than mice, in an attempt to reduce the potential effects of the age of the animals, the study was performed in *Peromyscus* about three times older than mice. Nevertheless, this difference was not expected to have major consequences on the tumorigenicity of the transplanted tumors.

**Fig. 1.**
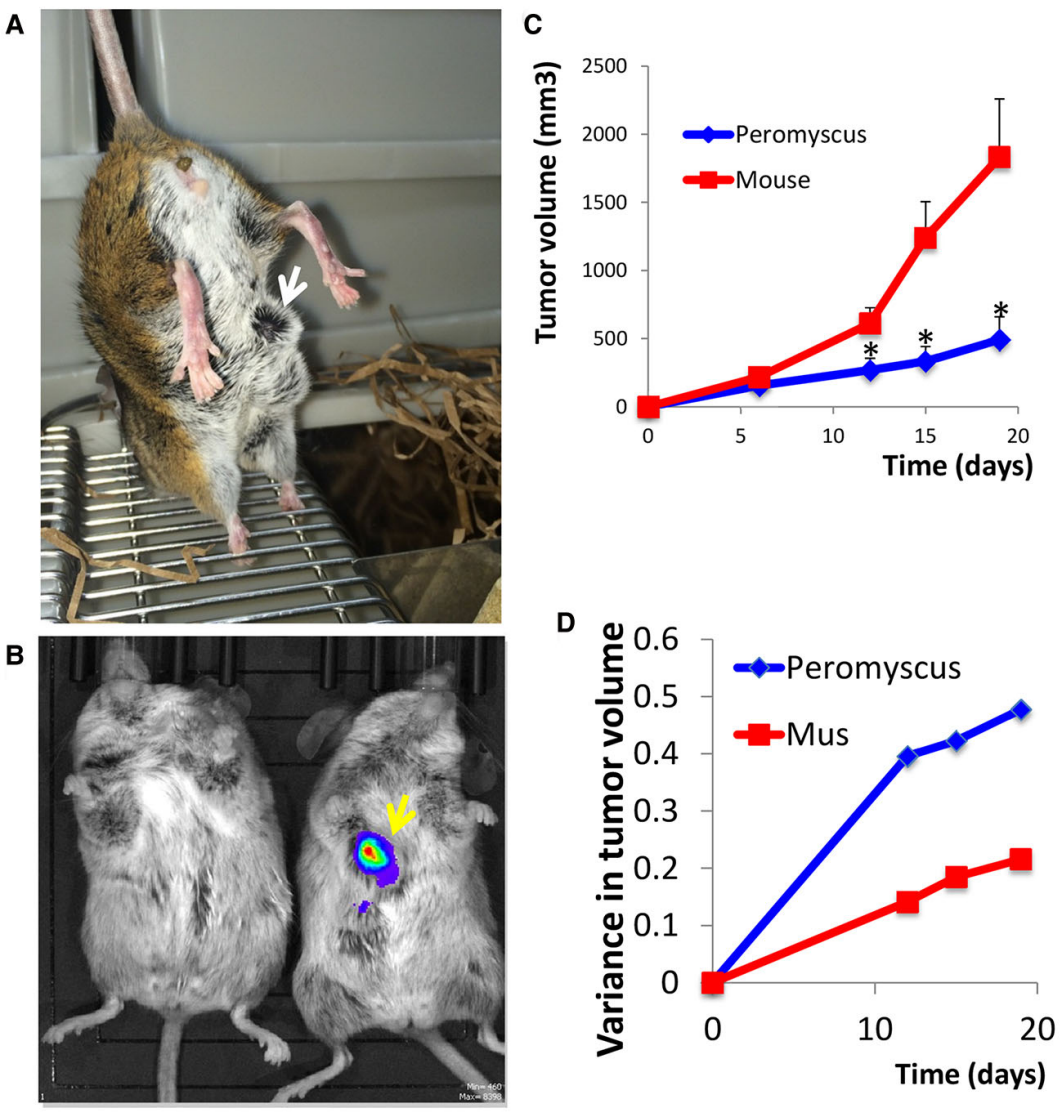
**MDA-MB-231 tumors in *P. californicus*.** (A) *P. californicus* bearing an MDA-MB-231 human breast tumor (arrow). (B) Whole-body imaging showing a tumor-free (left) and a luciferase-expressing MDA-MB-231 tumor-bearing (right) *P. californicus*. (C) Tumor growth rate of MDA-MB-231 admixed with human HFFF2 fibroblasts (1:1, 3 million each) and implanted in nude mice (*n*=5) or CsA-treated *P. californicus* (*n*=5). Results reflect average values±s.e.m. *P<0.05 Student's *t*-test. (D) Variance in normalized MDA-MB-231 tumor volumes, calculated by estimating the ratio between each individual tumor and the species' average for a given time point, and then by calculating the variance of these ratios.

**Fig. 2.**
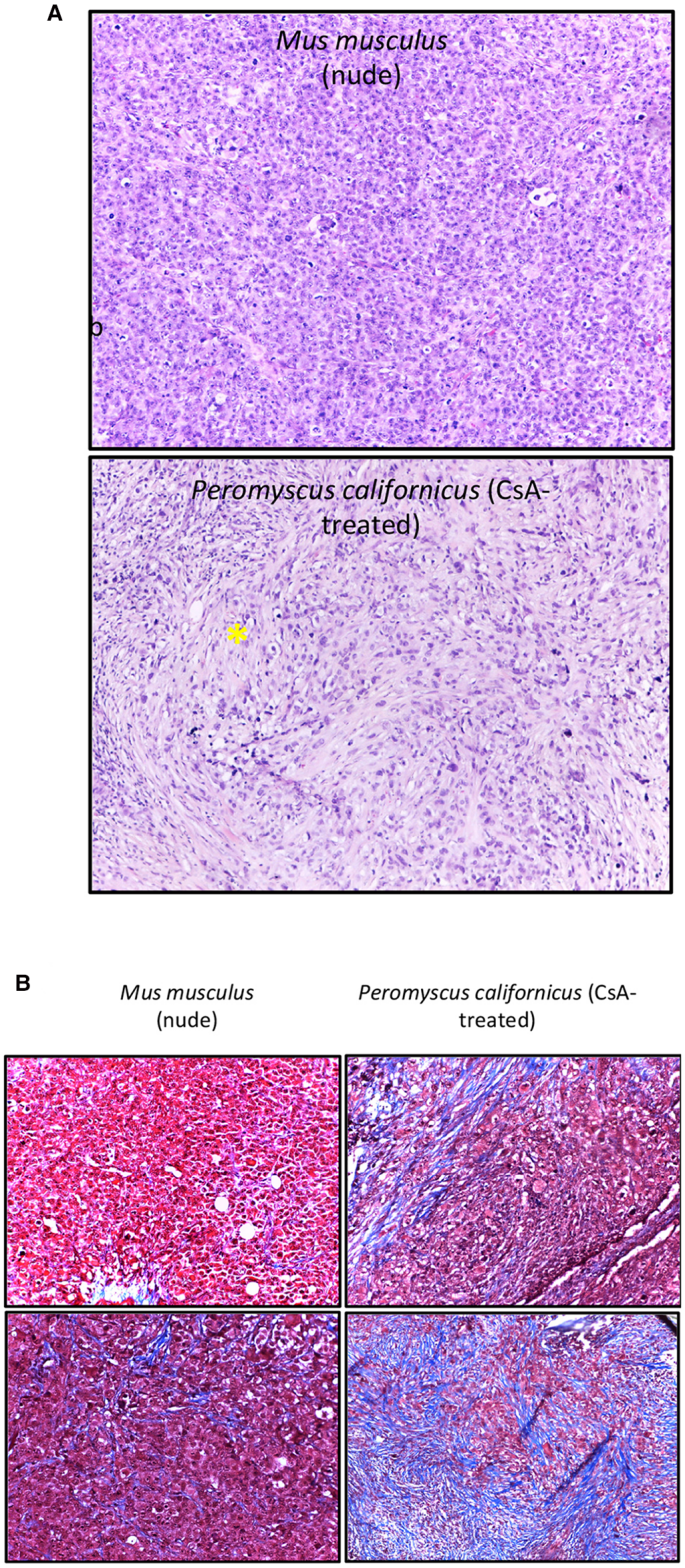
**Histology of MDA-MB-231 tumors.** (A) H&E-stained sections of tumors from MDA-MB-231 human breast cancer cells admixed with human HFFF2 fibroblasts in nude mice (upper panel) or *P. californicus* (lower panel). The asterisk indicates areas with spindling cells. Magnification, 10×. (B) Trichrome-stained sections of tumors from MDA-MB-231 human breast cancer cells admixed with human HFFF2 fibroblasts in nude mice (left) or *P. californicus* (right). Blue staining indicates the presence of collagen. Images from two tumors from each group are shown, selected to contain the lowest (top) and the highest (bottom) amounts of collagen. Magnification, 20×.

Our pilot study demonstrated that *P. californicus* efficiently supported the growth of MDA-MB-231 cells *in vivo*, whereas *P. leucopus* did not (data not shown). This observation is in line with the previously reported resistance of *P. leucopus* to urethane-induced carcinogenesis (Gross et al., 1953), and might be partially caused by the reduced mitogenic activity of the sera of *P. leucopus* as compared to *P. californicus* (Fig. S1). *P. californicus* is the largest *Peromyscus* species found in the United States (adult weight is ∼42 g), with a lifespan >5.5 years in captivity. This species is used extensively for behavioral studies of long-term monogamy and paternal care of young, as *P. californicus* naturally engages in both behaviors (Bester-Meredith et al., 2017). Whole-body imaging confirmed the presence of tumors in *P. californicus* (Fig. 1A,B). Although tumor growth rate in the *P. californicus* was reduced compared to that of nude mice, the rate of growth was more steady in the former (Fig. 1C). In nude mice, tumor growth rate increased considerably after about day 12, mandating termination of the experiment at day 20 because tumors exceeded 10% of the body weight. In addition, higher variance in tumor volumes was recorded for *Peromyscus* as compared to *Mus*, consistently with the outbred nature of the former as compared to the latter (Fig. 1D). Histologically, in both species, tumors were undifferentiated with marked cellular and nuclear pleomorphism and several mitotic figures. In addition, in the tumors formed in *P. californicus*, several areas with spindle cells were observed. This reflects the appearance of a desmoplastic stroma and simulates the histology of human breast cancers (Fig. 2A). To verify this evidence, we preformed trichrome staining (Masson) and showed that tumors in *P. californicus* had higher variability as compared to those in nude mice. We observed increased stroma and several areas with collagen fibers intermingling between the cancer cells in the tumors in *P. californicus* (Fig. 2B), while the tumors in nude mice had minimal stroma, and sparse collagen fibers present mostly at the periphery of the lesions. Furthermore, these results show that CsA administration in *Peromyscus* enhances, rather than eliminates, the stroma, as compared to conventional xenografts in which stroma is literally absent. Repetition of this experiment in wild-type (wt) C57B6 mice (CsA administration and implantation of MDA-MB-231 cells admixed with fibroblasts as described above) showed that stromal fibroblasts were absent from the tumors that occasionally had extensive necrotic areas (Fig. S2). These observations demonstrate a closer resemblance of *P. californicus* tumors with human primary breast cancers, which typically show involvement of the desmoplastic stroma.

### MCF-7 tumor growth

**Fig. 3.**
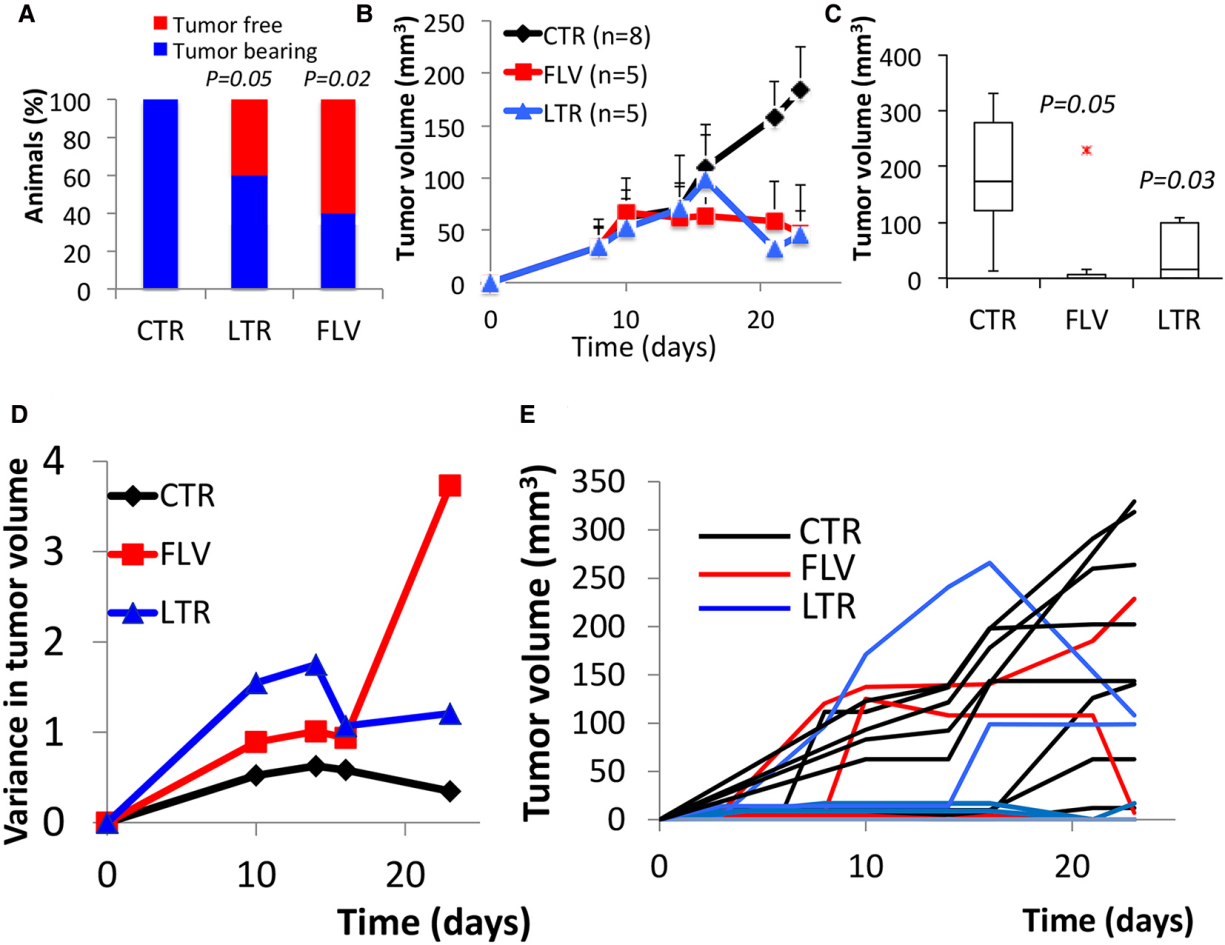
**MCF7 tumors in *P. californicus*.** (A-C) Tumor onset by day 23 (A), tumor growth rate (B) and final tumor volume (C) after inoculation of admixtures of MCF7/HFFF2 cells (1:1, 3 million each) in *P. californicus* treated with fulvestrant (FLV), letrozole (LTR) or DMSO (CTR). (D) Variance in normalized MCF7 control, FLV-treated or LTR-treated tumor volumes, calculated by estimating the ratio between each individual tumor and the group's average for a given time point, and then by calculating the variance of these ratios. (E) Growth of individual MCF7 tumors in mice treated with LTR or FLV. *P* values (Student's *t*-test) are indicated.

**Fig. 4.**
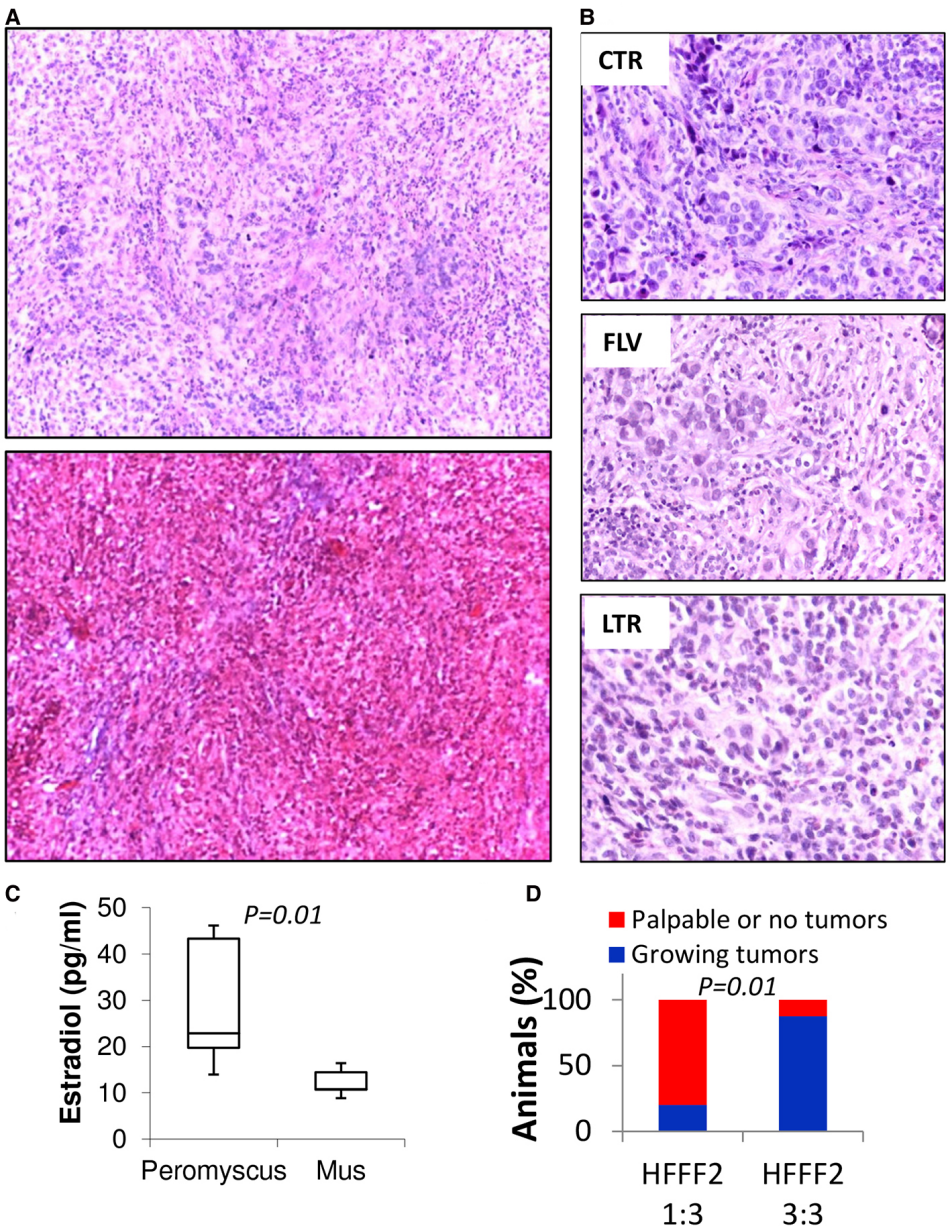
**Histology of MCF7 tumors in *P.*** ***californicus*****.** (A) H&E (top) or trichrome (bottom)-stained sections of MCF7 tumors in *P. californicus* (magnification, 10×). (B) Comparison of H&E stained sections of MCF7 tumors treated with FLV, LTR or DMSO (magnification, 40×). (C) Estradiol levels in the plasma of eight *P. californicus* and five female SCID mice measured by ELISA (Cayman Chemical, MI). *P* values are indicated (Student's *t*-test). (D) Percentage of tumors growing in relation to the ratio of HFFF2 fibroblasts admixed with the MCF7 cells. Tumors were scored as positive when tumor volume reached at least 100 mm^3^ after 3 weeks.

After identifying *P. californicus* as an appropriate host for breast tumor growth, we explored whether estrogen receptor-positive [ER (+)] human breast cancers also grow in these animals. Thus, we proceeded with tumorigenesis experiments using the widely used human estrogen-dependent MCF7 breast cancer cells, which in mice require E2 supplementation for adequate growth (Shafie, 1980; Huseby et al., 1984). We inoculated 9- to 12-month-old *P. californicus* (*n*=18) with MCF7 cells admixed with HFFF2 cells at a 1:1 ratio. After 1 week, about half of the animals presented with tumors, and treatment was initiated after equal distribution of tumor-bearing and tumor-free animals in the experimental groups. Of the 18 animals, eight received CsA only. Of the remaining 10, five received fulvestrant (1 mg/day) and five received letrozole (10 μg/day). These drugs represent the most commonly used monotherapies for the management of hormone-sensitive breast cancer in women (Lee et al., 2017; Kümler et al., 2016). Of the animals that were treated with CsA alone, all eight developed palpable tumors as opposed to only two of five (*P*=0.01), and three of five (*P*=0.05), animals that were treated with fulvestrant or letrozole, respectively (Fig. 3A). Tumor growth was significantly inhibited by both hormonal therapies, which in turn suggests that sensitivity to E2 had been retained by the MCF7 cells during their growth *in vivo* (Fig. 3B,C). Not surprisingly, a high variance was recorded in the growth profile of all MCF7 tumors that was enhanced in the treated groups, implying heterogeneity in the response, and is in line with the outbred nature of *Peromyscus* (Fig. 3D,E). Importantly, overall tumor growth inhibition was in the range of 70-75% for both treatments, exceeding by far the moderate tumor growth inhibition attained by these monotherapies in conventional laboratory mice (Fig. 3B). Histologically, tumors had a medullary-like appearance as they consist of anastomosing sheets of highly atypical cancer cells with several occasionally atypical mitoses and no obvious fibrosis of the stroma. The cancer cells were intermixed with a population of inflammatory cells including lymphocytes and neutrophils (Fig. 4A). Positivity to trichrome staining was reduced, as compared to the tumors developed by the MDA-MB-231 cells, indicating differential ability between these cell lines to mobilize the stroma (Fig. 4A). In the tumor sections from animals treated with fulvestrant and letrozole, and especially in those from animals treated with letrozole, a decrease in the number of cancer cells was observed (Fig. 4B).

Assessment of plasma E2 levels in eight *P. californicus* and five female SCID mice (aged 9-12 months and 4-5 months, respectively) showed that in the former they ranged between 13.9 pg/ml and 44.7 pg/ml, while in the latter they ranged between 8.9 pg/ml and 16.4 pg/ml, values that are close to the assay's detection limit of 15 pg/ml (average values for each species are 29.5±13.3 and 12.2±3.1, respectively, *P*=0.02, Student's *t*-test) (Fig. 4C). Thus, E2 is increased in *Peromyscus* but only moderately as compared to the levels in *Mus*, approximating the levels recorded in postmenopausal women (Geisler et al., 2002) and implying that E2 levels might not be the major determinant for the efficiency of tumorigenesis in *P. californicus*. Evidence that stromal cells might play an instrumental role in promoting tumor growth in this model has been provided by a different experiment in which MCF7 cells and HFFF2 fibroblasts were implanted at a ratio of 3:1. Under these conditions only one of five animals developed a growing tumor as opposed to seven of eight that had been implanted with MCF7:HFFF2 at a 1:1 ratio (*P*=0.01) (Fig. 4D). Thus, we conclude that for the growth of ER (+) breast cancer, the moderately elevated E2 levels in *Peromyscus* provide a permissive environment, but stromal cells drive tumor growth and can compensate for reduced endogenous E2.

## DISCUSSION

In the absence of a more suitable model, anti-estrogens are conventionally tested in animal models supplemented with E2. In particular, aromatase inhibitors are assessed *in vivo* in animals receiving androgen substrates, and by using genetically modified breast cancer cells engineered to overexpress and depend on aromatase for their growth. These pharmacological and genetic interventions present various limitations that restrict the value of the corresponding animal models. Aside from toxicity and maintenance of supraphysiological levels of estrogens, a direct comparison between different classes of anti-estrogens – such as aromatase inhibitors and estrogen receptor inhibitors – is not feasible unless the cancer cells have been modified to overexpress aromatase (Jelovac et al., 2004). By relying on endogenous estrogens, *P. californicus* overcomes these limitations, providing a model in which hormone-sensitive breast cancers can be studied and drugs can be assessed in a physiological context comparable to that of human patients.

Recent developments in breast tumor biology allowed the development of breast cancer models without E2 supplementation, based on the intraductal injection of breast cancer cells (Sflomos et al., 2016; Richard et al., 2016). However, these models require delicate experimental methodologies and severely immunocompromised mice. The proposed here outbred model has the advantage of genetic heterogeneity, which facilitates studies exploring tumorigenesis and anticancer drug efficacy in naturally existing populations. The increased variance in the response to letrozole and fulvestrant between different animals, as compared to the variance of untreated controls, reflects the power of this experimental system and confirms that variation in the efficacy of hormone therapy is independent from the intrinsic growth ability of individual tumors. More striking is the fact that this variability in the antitumor activity of both fulvestrant and letrozole is not caused by molecular heterogeneity of the cancer cells but rather by heterogeneity of the host. This in turn implies that focused analyses of cancer cells, especially *in vitro*, for the revelation of mechanisms of resistance is, in many cases, clinically irrelevant.

Another advantage of this model is relevant to the fact that *P. californicus* lives almost three times longer than *Mus*. This allows the design of long-term studies before the experimental animals age, and facilitates the investigation of breast cancer biology and therapy in clearly defined, aged distinct experimental groups.

Furthermore, our results suggest that even low endogenous levels of E2 generate a permissive environment for mammary tumorigenesis, and are adequate to support the growth of ER (+) tumors. The fact that increasing numbers of stromal cells drastically enhanced the tumorigenicity of MCF7 cells underscores the contribution of the microenvironment, and suggests that tumor promotion by stromal activation might partially substitute the reduced estrogen stimulation. Indeed, the importance of the stromal fibroblasts in the profile of tumorigenesis and histopathology has been demonstrated (Kiaris et al., 2005; Lafkas et al., 2008). This observation could also have implications related to the fact that in postmenopausal women, estrogen levels, albeit not cycling, are closer to the levels of E2 recorded in *P. californicus*.

Collectively, our results suggest that, through appropriate genetic manipulation or optimization of CsA-mediated immunosuppression, *P. californicus* might provide an attractive alternative to mice for modeling breast cancer in genetically heterogenous hosts. This model can be especially useful in studying ER (+) breast cancers *in vivo*, without estrogen supplementation. Besides its value in studying basic aspects of estrogen-dependent tumor biology, this model could find application in the study of testing anti-estrogen-based therapeutics and the onset of drug resistance.

## MATERIALS AND METHODS

### Cell culture

MDA-MB-231/Luc, MCF7 and HFFF2 cell lines were cultured in Dulbecco's modified Eagle medium (DMEM) with 10% fetal bovine serum (Corning). HFFF2 cells were obtained as cryopreserved cells from Sigma-Aldrich and were used in all assays between passage five and ten. MDA-MB-231/Luc cells were obtained from Cell Biolabs, and MCF7 cells from ATCC (Manassas, VA) and subsequently maintained in our laboratory. MDA-MB-231 and MCF7 cells were authenticated prior to *in vivo* studies by short-tandem repeat (STR) analysis (Biosynthesis, Lewisville, TX and University of Arizona Genetic Core, Tucson, AZ, respectively). All cell lines were frequently tested for mycoplasma contamination using a commercially available Mycoplasma detection kit (Myco Alert kit, Lonza). Cell proliferation studies were performed in triplicate as described (Farmaki et al., 2016; Porter et al., 2012).

### Animal studies

*P. californicus* and *P. leucopus* were obtained from the Peromyscus Genetic Stock Center (Columbia, SC). NCr nude mice were obtained from Taconic. C57B6 mice were originally obtained from the Jackson Laboratory and maintained at the University of South Carolina. Animals at the age indicated were treated with CsA (in 90% olive oil and 10% EtOH) s.c. at 50 mg/kg daily. Cancer cells and fibroblasts were implanted orthotopically in the mammary fat pad of the third thoracic mammary gland. Animals were euthanized under isoflurane anesthesia. Animal studies complied with Institutional guidelines. For tumor reconstitution experiments, cells (at numbers described in the Results section), were re-suspended in 0.2 ml serum-free DMEM and then injected s.c. into the mammary fat pad. Tumor volumes were assessed by using the formula (width)^2^×length/2. All experiments were approved by the Institutional Animal Care and Use Committee of the University of South Carolina. Fulvestrant and letrozole were obtained from Sigma-Aldrich and were dissolved initially in DMSO and then combined in an emulsion with CsA in 90% olive oil and 10% EtOH, and administered daily s.c. at 1 mg/day and 10 μg/day.

### Histology

For histological analyses, tumors were fixed in 10% formalin, paraffin-embedded, serially sectioned, and stained with Hematoxylin/Eosin (H&E). Trichrome staining was performed using a kit from Abcam (Cambridge, MA), following the manufacturer's instructions. Images shown were obtained by a Leica ICC50 HD.

### Statistics

All data are presented as average values of samples, error bars correspond to standard error of the mean (s.e.m.) unless otherwise stated. Statistical analysis of the results was performed using Student's two-tailed *t*-test. The results were considered statistically significant when *P*<0.05.

## Supplementary Material

Supplementary information
