# Supplementary material for: Growth of human breast cancers in Peromyscus
Source: Dis Model Mech. 2018 Jan 1;11(1):dmm031302. doi: 10.1242/dmm.031302 (PMC5818077; doi:10.1242/dmm.031302)
Supplement: Supplementary information [file dmm-11-031302-s1.pdf]

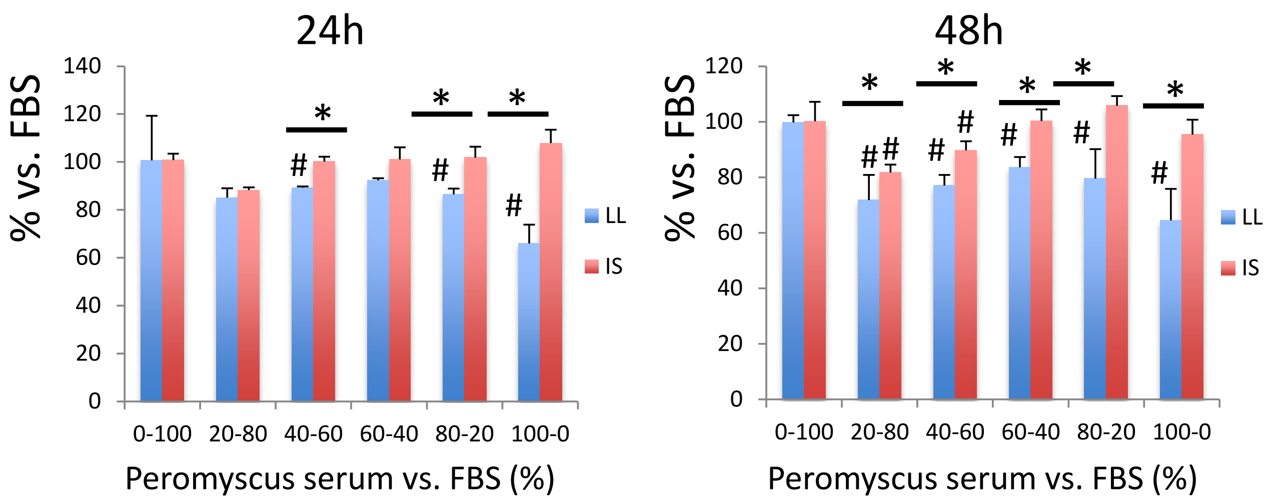

Supplementary Figure 1. Growth of MDA-MB-231 cells in the presence of different concentrations of Peromyscus (LL, *P. leucopus*; IS, *P. californicus*) serum mixed with FBS. For this experiment sera from the corresponding *Peromyscus* species were mixed with FBS at the indicated ratios and their effects in MDA-MB-231 cell proliferation was assessed after 24h and 48h. Results are expressed as average of triplicates ± SEM. *, P<0.05; #, P<0.05 vs. FBS.

Disease Models & Mechanisms • Supplementary information

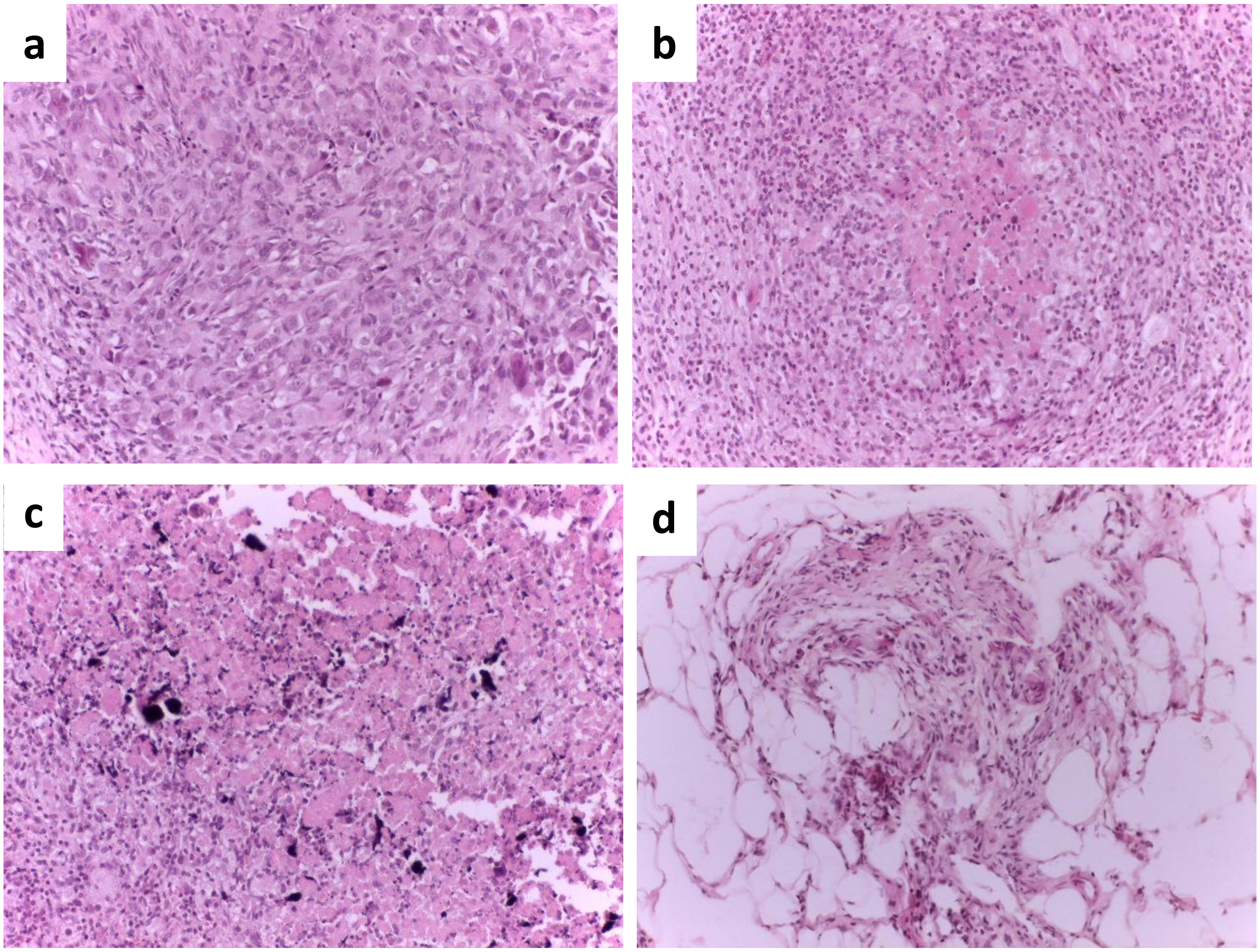

Supplementary Figure 2. Growth of MDA-MB-231 cells in C57B6 mice immunosuppressed by CsA. In Mus tumors became palpable in 4 out 5 animals, 4 days after cancer cell implantation and that tumors were largely deficient of stroma. Only in one out 4 cases an aggressive adenocarcinoma was detected (a), while 2 tumors were highly necrotic (b and c) and the forth was a scar tissue with minimal neoplastic lesions (c).
